# EZH2 Expression and Clinical Outcomes in Non-Small Cell Lung Cancer Patients Treated with Immune Checkpoint Inhibitors: A Real-World Retrospective Cohort Study

**DOI:** 10.3390/jcm15176611

**Published:** 2026-08-27

**Authors:** Esra Asarkaya, Hatice Asoglu, Abdurrahman Aykut, Gunes Dorukhan Cavusoglu, Yasemin Aydınalp, Sendag Yaslıkaya, Suheda Atas Ipek, Fatma Calkan, Emine Kilic Bagir, Derya Gumurdulu, Hulya Binokay, Tolga Koseci, Ismail Oguz Kara, Berksoy Sahin, Ertugrul Bayram

**Affiliations:** 1 Department of Medical Oncology, Cukurova University Faculty of Medicine, 01330 Adana, Türkiyedr.abdurrahmanaykut@gmail.com (A.A.); gunescavusoglu@gmail.com (G.D.C.); suhedaatas92@gmail.com (S.A.I.); drtolgakoseci@gmail.com (T.K.); ioguzkara@gmail.com (I.O.K.); berksoys@hotmail.com (B.S.); ertugrulbayram84@gmail.com (E.B.); 2 Department of Medical Oncology, Sanlıurfa Mehmet Akif İnan Training and Research Hospital, 63300 Şanlıurfa, Türkiye; yaseminaydinalp23@gmail.com; 3 Department of Medical Oncology, Giresun Training and Research Hospital, 28100 Giresun, Türkiye; drysendag@gmail.com; 4 Department of Pathology, Cukurova University Faculty of Medicine, 01330 Adana, Türkiye; calkanfatma@gmail.com (F.C.); eminebagir@yahoo.com (E.K.B.); gumurdulu@yahoo.com (D.G.); 5 Department of Biostatistics, Cukurova University Faculty of Medicine, 01330 Adana, Türkiye; hulyabinokay@gmail.com

**Keywords:** EZH2, NSCLC, immune checkpoint inhibitors

## Abstract

**Background:** Lung cancer remains the leading cause of cancer-related mortality, with non-small cell lung cancer (NSCLC) accounting for approximately 85% of cases. Over the past decade, immune checkpoint inhibitors have become a core component of first-line treatment for advanced-stage NSCLC lacking driver mutations. Programmed death-ligand 1 (PD-L1) expression is currently used as the standard biomarker, yet its predictive value remains limited, and in most immunotherapy trials, treatment efficacy has been observed independently of PD-L1 expression status. Enhancer of zeste homolog 2 (EZH2), an epigenetic regulator, promotes immune escape by suppressing antigen presentation and impairing CD8+ T-cell function, thereby generating an immune-cold tumor microenvironment, positioning it as a promising candidate biomarker. **Methods:** We retrospectively analyzed 102 NSCLC patients treated with immunotherapy at a single center between 2018 and 2024. EZH2 expression was assessed by immunohistochemistry. A cohort-derived 25% threshold was used for the primary exploratory analysis, and the analyses were repeated using a 50% threshold as a sensitivity analysis. Patients were classified as EZH2-high (45.1%) and EZH2-low (54.9%) at the 25% threshold. **Results:** At the 25% threshold, objective response rate (ORR) was 53.6% in the EZH2-low group and 45.7% in the EZH2-high group (Fisher’s exact *p* = 0.551), while disease control rate (DCR) was 64.3% and 60.9%, respectively (*p* = 0.837). Median overall survival (OS) was 37 versus 27 months (log-rank *p* = 0.323), and median progression-free survival (PFS) was 15 versus 12 months (*p* = 0.387). No significant correlation was found between EZH2 and PD-L1 expression (r = 0.167, *p* = 0.138). In treatment-line-adjusted Cox models, EZH2 expression was not associated with OS (hazard ratio (HR) 0.953, 95% confidence interval (CI) 0.533–1.704; *p* = 0.870) or PFS (HR 0.996, 95% CI 0.573–1.733; *p* = 0.989), whereas squamous histology was an independent predictor of survival. Results remained non-significant at the 50% threshold. Early progression was uncommon and did not differ significantly by EZH2 status overall or within PD-L1 strata. **Conclusions:** In this real-world cohort, EZH2 expression was not independently associated with response, early progression, OS, or PFS, and showed no correlation with PD-L1. These exploratory findings do not support the clinical use of EZH2 as a biomarker at this stage; prospective, multicenter studies using predefined thresholds and standardized immunohistochemical methods are needed to clarify its potential role as a marker complementary to PD-L1.

## 1. Introduction

Approximately 2 million new lung cancer cases are diagnosed worldwide each year, and 1.76 million people die from the disease, making it the leading cause of cancer-related mortality [1]. The introduction of immune checkpoint inhibitors into clinical practice has fundamentally transformed NSCLC treatment over the past decade; these agents have now become the first-line standard of care, either as monotherapy or in combination with chemotherapy, for patients with advanced-stage disease lacking driver mutations [2,3]. Nevertheless, both primary and acquired resistance remain a major clinical challenge, and a substantial proportion of patients fail to derive meaningful benefit from immunotherapy.

Although programmed death-ligand 1 (PD-L1) remains the most widely used biomarker for predicting response to immune checkpoint inhibitors, it is increasingly evident that this marker alone does not provide sufficient discriminatory power in clinical practice. In two phase III trials (CheckMate 017 and CheckMate 057), nivolumab demonstrated improved overall survival compared with docetaxel in previously treated patients with advanced squamous and non-squamous NSCLC, an effect observed regardless of PD-L1 expression level [4]. This is consistent with current international guideline recommendations, which support first-line chemoimmunotherapy and second-line or later single-agent immune checkpoint inhibitor use irrespective of PD-L1 expression status [5]. Moreover, even within the most highly selected patient subgroup—those with PD-L1 expression ≥ 50%—response to immunotherapy remains limited: objective response rates reported in phase III trials for pembrolizumab, atezolizumab, and cemiplimab were 44.8%, 40.2%, and 46.5%, respectively, indicating that more than half of patients fail to respond [6,7,8]. These findings underscore the limited predictive value of PD-L1 expression and highlight the critical need for additional biomarkers to better identify patients likely to benefit from immunotherapy. In this context, molecules that shape the tumor microenvironment and immune response at the epigenetic level have emerged as promising candidate biomarkers.

Epigenetic modifications are defined as heritable phenotypic changes that do not arise from alterations in the primary DNA sequence [9]. EZH2 is the core methyltransferase of the polycomb repressive complex 2 (PRC2) and catalyzes H3K27me3 modification, which silences tumor suppressor genes such as p16, CDH1, and PTEN and thereby promotes cell proliferation, epithelial–mesenchymal transition, invasion, and metastasis. EZH2 overexpression has been reported in a wide range of malignancies, including breast, ovarian, and prostate cancer, and has been associated with aggressive disease behavior [10]. The role of EZH2 is not limited to carcinogenesis; it has also been shown to contribute to resistance against platinum-based therapy in ovarian cancer and against androgen pathway blockade in prostate cancer [11,12]. EZH2 overexpression is also frequently observed in lung cancer, where its effects extend beyond its oncogenic role to include several immunoregulatory mechanisms within the tumor microenvironment. It suppresses MHC-I expression and T-cell chemokines such as CXCL9 and CXCL10, limiting antigen presentation and CD8+ T-cell infiltration [13]; at the same time, methylation of cytotoxic factor genes such as IFNG and GZMB helps maintain the immunosuppressive phenotype of Treg cells, weakening the effector function of CD8+ T cells [14]. These combined effects on intercellular signaling within the tumor microenvironment may drive immunotherapy resistance and disease progression. Beyond these microenvironmental effects, a more direct link between EZH2 and PD-L1 has also been reported: increased EZH2 expression in lung cancer tissue has been found to be associated with higher PD-L1 levels. In the study by Zhao et al., silencing of EZH2 reduced PD-L1 expression through a decrease in hypoxia-inducible factor-1α (HIF-1α), which activates the PD-L1 promoter [15].

Here, we evaluated the relationship between EZH2 expression and disease control and survival in patients with non-small cell lung cancer treated with immune checkpoint inhibitors.

## 2. Materials and Methods

### 2.1. Study Design and Patients

This retrospective, single-center real-world study included 102 patients with histologically confirmed non-small cell lung cancer (NSCLC) who were treated with immune checkpoint inhibitors (ICIs) between January 2018 and December 2024 at the Medical Oncology Department of Cukurova University Faculty of Medicine. Eligible patients were required to have available clinical and follow-up data as well as adequate tumor tissue for immunohistochemical evaluation. Patients with incomplete records, insufficient tissue samples, or known oncogenic driver mutations (including EGFR, ALK, ROS1, and other actionable alterations) were excluded from the analysis.

### 2.2. Data Collection

Clinical and clinicopathological data were retrieved from institutional electronic medical records. Variables collected for each patient included age at diagnosis, sex, histological subtype, stage at diagnosis, smoking status, line of immunotherapy, type of ICI regimen, and prior treatments. Survival status and follow-up duration were recorded for all patients.

### 2.3. Biomarker Assessment

#### 2.3.1. PD-L1 Expression

PD-L1 expression was assessed using the Dako 22C3 pharmDx assay (Dako, Glostrup, Denmark). Tumor proportion score (TPS) was used for evaluation and defined as the percentage of viable tumor cells showing membranous staining. Patients were stratified as PD-L1-negative (TPS < 1%) and PD-L1-positive (TPS ≥ 1%), with additional subgroup analyses performed in patients with TPS ≥ 50%.

#### 2.3.2. EZH2 Expression

EZH2 expression was assessed by immunohistochemistry (IHC) on formalin-fixed, paraffin-embedded tumor tissue samples obtained at diagnosis. Sections were stained using a mouse monoclonal EZH2 (clone 11) antibody (Cell Marque, Rocklin, CA, USA; Sigma-Aldrich, St. Louis, MO, USA; Ref. 415M-15) at a 1:75 dilution, with staining performed on a Ventana BenchMark automated staining system and antigen retrieval carried out using Cell Conditioning 1 (CC1) solution followed by 64 min incubation with the primary antibody and visualized with the Ventana DAB substrate kit (cat. no. 957D-20; Roche Diagnostics, Basel, Switzerland). Nuclear staining was considered indicative of EZH2 positivity. The percentage of tumor cells showing nuclear staining was recorded, and staining intensity was evaluated accordingly. All slides were independently reviewed by three experienced pathologists blinded to clinical outcomes; discrepancies were resolved by consensus through joint review. Independent pre-consensus scores were not retained, precluding a formal interobserver reliability estimate. Patients were stratified into high (≥25%) and low (<25%) EZH2 expression groups; ≥50% versus <50% was evaluated in sensitivity analyses. The 25% cut-off was derived using multivariable Cox regression within the same study cohort and is therefore considered cohort-derived and exploratory rather than externally validated. At this threshold, patients were classified as EZH2-high (45.1%) and EZH2-low (54.9%).

### 2.4. Treatment and Response Evaluation

All patients received ICIs as part of routine clinical practice, either as monotherapy or in combination with chemotherapy, depending on clinical indication and treatment line. Chemoimmunotherapy was used as the preferred first-line regimen regardless of PD-L1 expression status, and single-agent ICI was used in the second-line setting and beyond, independent of PD-L1 status, consistent with international clinical practice guidelines. Tumor response was assessed according to Response Evaluation Criteria in Solid Tumors (RECIST) version 1.1. Responses were categorized as complete response (CR), partial response (PR), stable disease (SD), or progressive disease (PD). Objective response rate (ORR) was defined as the proportion of patients achieving CR or PR, while disease control rate (DCR) was defined as the proportion achieving CR, PR, or SD.

### 2.5. Outcomes

Progression-free survival (PFS) was defined as the time from initiation of immunotherapy to documented disease progression or death from any cause. Overall survival (OS) was defined as the time from initiation of immunotherapy to death or last follow-up. Early progression was defined as disease progression occurring within the first three months of treatment initiation.

### 2.6. Statistical Analysis

Statistical analyses were performed using SPSS software (version 20; IBM Corp., Armonk, NY, USA). Categorical variables were summarized as frequencies and percentages. All percentages for response and disease control used the total number of patients within each EZH2 group as the denominator. Group comparisons used the chi-square test or two-sided Fisher’s exact test, as appropriate. Survival curves were estimated using the Kaplan–Meier method and compared using the log-rank test. The correlation between continuous EZH2 and PD-L1 expression was assessed using Pearson’s correlation coefficient. The cohort-derived 25% EZH2 threshold was treated as exploratory; all response, early-progression, OS, PFS, and multivariable Cox analyses were repeated using 50% as a sensitivity threshold. Multivariable Cox models for OS and PFS included EZH2 group, ECOG performance status, histological subtype, PD-L1 status, and immunotherapy line (first, second, or third). Cox analyses used complete cases because PD-L1 status was unavailable for 21 patients. Exploratory early-progression comparisons were additionally assessed with a Bonferroni correction across the overall, second-line, PD-L1-positive, and PD-L1-negative analyses. A two-sided *p*-value < 0.05 was considered statistically significant.

### 2.7. Ethics Approval

The study was conducted in accordance with the Declaration of Helsinki and approved by the Çukurova University Non-Interventional Clinical Research Ethics Committee (approval date: 26 July 2024; meeting no: 146; decision no: 57). Due to the retrospective design, the requirement for informed consent was waived.

## 3. Results

A total of 102 patients were included in the study. The median age was 63 years (range, 41–82), and 86.3% of patients were male. The most common histological subtype was adenocarcinoma (52.9%), and 65.7% of patients presented with metastatic disease at diagnosis. Baseline characteristics are summarized in Table 1.

Of the patients, 17.6% (*n* = 18) received immunotherapy as first-line treatment, 72.5% (*n* = 74) as second-line, and 9.8% (*n* = 10) as third-line treatment. Among the 18 patients who received first-line treatment, 11 (61.1%) were treated with chemoimmunotherapy and the remaining 7 with immunotherapy alone; the overall rate of chemoimmunotherapy in the cohort was 10.8%. All patients receiving second- and third-line treatment were treated with immunotherapy alone. Nivolumab was the most commonly used agent. Thoracic palliative radiotherapy was administered in 31.4% of patients. Detailed treatment characteristics are summarized in Table 2.

**Figure 1:**
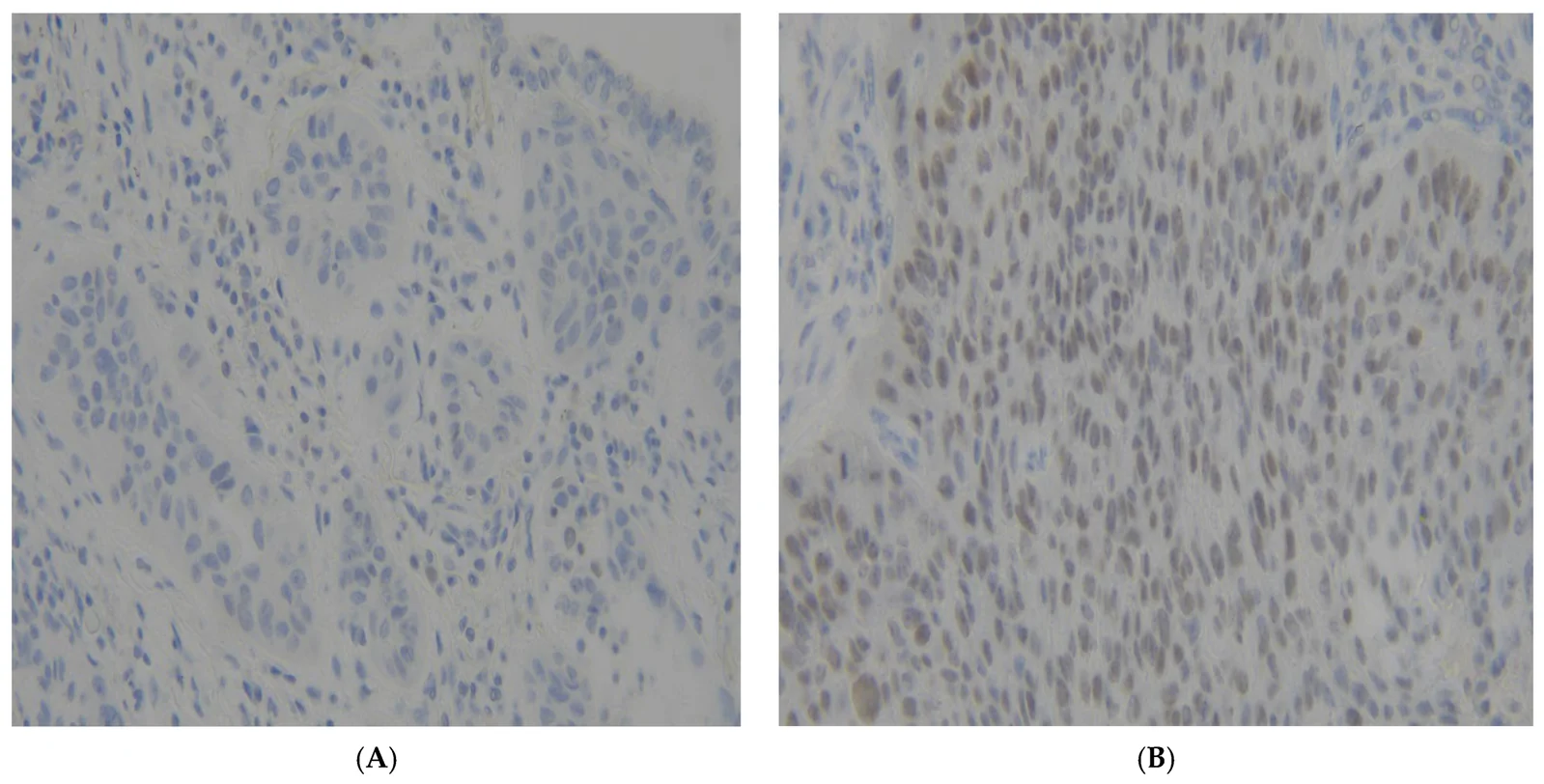
Representative immunohistochemical staining of EZH2 in tumor samples from patients with non-small cell lung cancer. (**A**) Low EZH2 expression (<25% nuclear staining). (**B**) High EZH2 expression (≥25% nuclear staining). Original magnification ×200.

In our cohort, EZH2 expression was low (<25%) in 56 patients (54.9%) and high (≥25%) in 46 patients (45.1%). Representative immunohistochemical images of EZH2 expression are shown in Figure 1.

Objective response rate (ORR) was 53.6% in the EZH2-low group (30/56) and 45.7% in the EZH2-high group (21/46; Fisher’s exact *p* = 0.551). Disease control rate (DCR) was 64.3% (36/56) and 60.9% (28/46), respectively (Fisher’s exact *p* = 0.837). The corresponding 2 × 2 counts and group-specific denominators are shown in Table 3.

At the 25% threshold, Kaplan–Meier analysis showed no statistically significant difference between the EZH2-low and EZH2-high groups in OS (median 37 months, 95% CI 20–81, vs. 27 months, 95% CI 16–55; log-rank *p* = 0.323) or PFS (median 15 months, 95% CI 10–24, vs. 12 months, 95% CI 7–30; *p* = 0.387). At the 50% sensitivity threshold, median OS was 33 months (95% CI 20–61) in the low group and 26 months (95% CI 9–55) in the high group (*p* = 0.228); median PFS was 15 months (95% CI 12–24) and 9 months (95% CI 4–37), respectively (*p* = 0.295).

**Figure 2:**
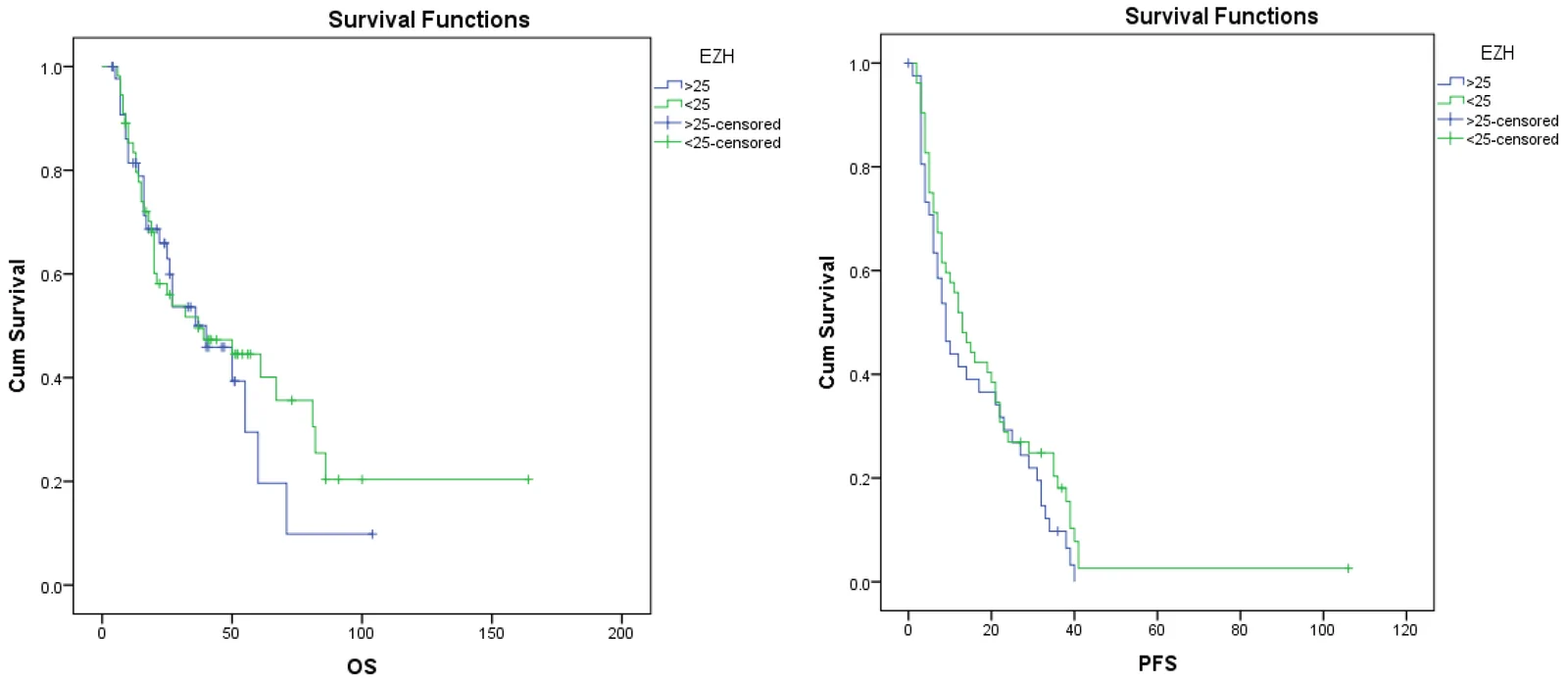
Kaplan–Meier survival curves for overall survival (OS) and progression-free survival (PFS) according to EZH2 expression status. Log-rank *p* = 0.323 for OS; *p* = 0.387 for PFS.

Kaplan–Meier survival curves for OS and PFS according to EZH2 expression are shown in Figure 2.

Using the prespecified operational definition of a documented PFS event within three months, early progression occurred in 8/46 (17.4%) EZH2-high and 4/56 (7.1%) EZH2-low patients (OR 2.737, 95% CI 0.768–9.756; Fisher’s exact *p* = 0.132). In the second-line subgroup, early progression occurred in 7/34 (20.6%) versus 3/40 (7.5%), respectively (OR 3.198, 95% CI 0.757–13.504; *p* = 0.171). Neither comparison was statistically significant, and the Bonferroni-adjusted *p*-values were 0.526 and 0.684, respectively.

**Figure 3:**
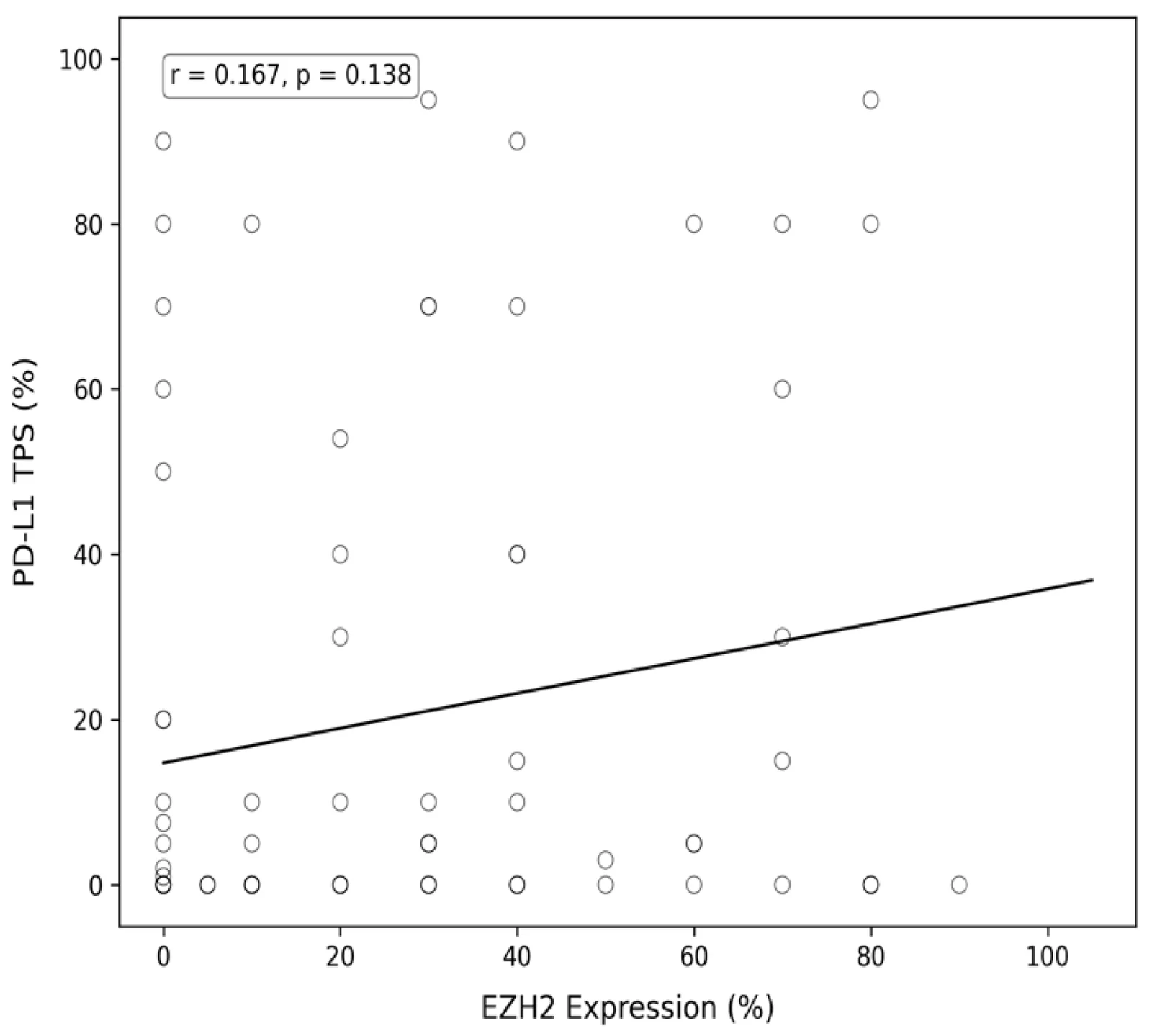
Scatter plot illustrating the correlation between EZH2 expression (%) and PD-L1 tumor proportion score (%). No significant correlation was identified between the two biomarkers (r = 0.167, *p* = 0.138).

PD-L1 expression was evaluated using the tumor proportion score (TPS): 38 patients (37.3%) were classified as PD-L1-negative (TPS < 1%) and 43 patients (42.2%) as PD-L1-positive (TPS ≥ 1%), while PD-L1 data were unavailable for 21 patients (20.6%). The detailed TPS distribution is presented in Table 1. Objective response rate was 60.5% in PD-L1-positive patients (26/43) compared with 39.5% in PD-L1-negative patients (15/38) (*p* = 0.076); although this difference approached the threshold of statistical significance, it did not reach significance. No significant differences were observed between PD-L1-positive and PD-L1-negative groups in progression-free survival or overall survival on Kaplan–Meier analysis. No significant correlation was observed between EZH2 expression and PD-L1 expression status (r = 0.167, *p* = 0.138) (Figure 3).

Among PD-L1-positive patients (*n* = 43), early progression occurred in 4/24 (16.7%) EZH2-high and 1/19 (5.3%) EZH2-low patients (OR 3.600, 95% CI 0.368–35.266; Fisher’s exact *p* = 0.363). Among PD-L1-negative patients (*n* = 38), the corresponding rates were 1/13 (7.7%) and 3/25 (12.0%) (OR 0.611, 95% CI 0.057–6.537; *p* = 1.000). Both confidence intervals were wide, and both Bonferroni-adjusted *p*-values were 1.000. These subgroup findings are therefore considered exploratory (Table 4).

In our cohort, six patients (5.9%) achieved a deep and durable response with immunotherapy and were transitioned to treatment-free follow-up after two years of treatment. Among these exceptional responders, low EZH2 expression was observed in four patients. PD-L1 data were available for three of these patients; two were PD-L1-positive and one was PD-L1-negative. Because this descriptive subset contained only six patients, no association or predictive inference can be drawn; the observation is reported solely as hypothesis-generating in light of preclinical evidence that EZH2 inhibition may enhance antitumor immune responses.

Treatment-line-adjusted multivariable Cox analyses were performed in 81 complete cases with known PD-L1 status (52 OS events and 58 PFS events). At the 25% threshold, EZH2-high expression was not associated with OS (HR 0.953, 95% CI 0.533–1.704; *p* = 0.870) or PFS (HR 0.996, 95% CI 0.573–1.733; *p* = 0.989). Neither second- nor third-line treatment was independently associated with OS or PFS. Squamous histology remained associated with poorer OS (HR 2.580, 95% CI 1.370–4.857; *p* = 0.003) and PFS (HR 1.888, 95% CI 1.066–3.346; *p* = 0.029) relative to adenocarcinoma (Table 5). Sensitivity analyses at the 50% threshold yielded the same substantive conclusion (Table 6).

## 4. Discussion

In this real-world retrospective study, we evaluated the association between EZH2 expression and clinical outcomes in 102 patients with NSCLC treated with immune checkpoint inhibitors. Objective response rate, disease control rate, and median overall and progression-free survival were all numerically higher in the EZH2-low group, although none of these differences reached statistical significance at either the 25% or 50% threshold. In the literature, there were three independent meta-analyses investigating the relationship between EZH2 expression and prognosis in non-small cell lung cancer, comprising 34 studies with a total of 5807 patients. High EZH2 expression was found to be associated with worse survival (HR range: 1.65–1.94). In these studies, a universally accepted cut-off value for EZH2 expression had not been determined; the positivity rates used in individual studies ranged from 46% to 66% [16,17,18]. Only in the study by Cao et al. was it reported that the median-based threshold was around 50% [19]. Although these comparisons did not reach statistical significance, examination of the Kaplan–Meier curve for overall survival revealed a clear separation in favor of the EZH2-low group was observed from approximately month 40–50 onward. The relatively small sample size of the study may have limited the statistical power of our study. In addition, our cohort consisted of a heterogeneous population including different treatment lines (17.6% first-line, 72.5% second-line, 9.8% third-line) and different immunotherapy agents; this may have made it difficult to reach statistical significance.

In this study, EZH2 expression was not found to be an independent prognostic factor in multivariable Cox regression analysis. A recent single-center retrospective study by Karadurmuş et al. in lung carcinoma patients similarly found that EZH2 expression, despite frequent positivity, was not independently associated with overall survival in multivariable Cox regression [20]. This underscores that mechanistic plausibility at the cellular level does not necessarily translate into detectable clinical utility in unselected, real-world patient populations. In our study, 39.2% of patients had squamous histology, and squamous cell carcinoma was found to be an independent predictor for survival in multivariable analysis. Fan et al. reported that the prognostic effect of EZH2 was not significant in squamous cell carcinoma (HR 1.03, 95% CI 0.81–1.30; *p* = 0.820), and was significant only in adenocarcinoma (HR 1.27, 95% CI 1.01–1.60; *p* = 0.045) [18]. Notably, adenocarcinoma was the predominant histology in our cohort (52.9%), the subtype in which this effect was previously reported; the absence of an independent EZH2 association despite this histological composition suggests that the prognostic relevance of EZH2 may be more context-dependent than a single IHC threshold can capture.

In our cohort, the progression rate in the first 3 months of treatment was found to be numerically higher in patients with EZH2 overexpression compared to EZH2-low patients (17.4% vs. 7.1%; OR 2.737, 95% CI 0.768–9.756), although this difference did not reach statistical significance (Bonferroni-adjusted *p* = 0.526). A similar numerical trend was observed in the second-line subgroup, which was more homogeneous in terms of treatment (20.6% vs. 7.5%; OR 3.198, 95% CI 0.757–13.504; Bonferroni-adjusted *p* = 0.684). While these estimates do not establish EZH2 as a marker of early treatment resistance, biological plausibility exists: EZH2 reduces tumor immunogenicity by impairing antigen presentation through epigenetic silencing. In preclinical studies, it has been shown that adding an EZH2 inhibitor to anti-CTLA4 or IL-2 therapy in mouse melanoma models rendered the immunosuppressive tumor microenvironment immunoactive and increased immunotherapy efficacy [21]. In human ovarian cancer samples, EZH2 has been shown to suppress chemokine expression, and in mouse tumor models, EZH2 inhibition has been shown to render tumors sensitive to immunotherapy [22].

No significant correlation was found between EZH2 expression and PD-L1 status (r = 0.167, *p* = 0.138), suggesting that the two biomarkers may be based on different biological mechanisms. The existing literature on the EZH2-PD-L1 relationship remains limited and inconsistent across tumor types, underscoring the need for further investigation. When PD-L1 and EZH2 were considered together, no significant relationship was found between EZH2 expression and early progression in either PD-L1-positive patients (16.7% vs. 5.3%; OR 3.600, 95% CI 0.368–35.266; *p* = 0.363) or PD-L1-negative patients (7.7% vs. 12.0%; OR 0.611, 95% CI 0.057–6.537; *p* = 1.000). Both confidence intervals were wide, and these subgroup findings are therefore considered exploratory.

In our cohort, six patients (5.9%) were being followed without treatment due to the deep and durable response achieved after 2 years of treatment. Low EZH2 expression was found in four (66.7%) of these patients. Among the three patients with available PD-L1 data, two were PD-L1-positive and one was PD-L1-negative. Given the very small sample size and the absence of a comparator group, this observation cannot support a prognostic, predictive, or therapeutic inference regarding EZH2. It is reported only as hypothesis-generating, in light of evidence that combining EZH2 inhibitors with immunotherapy has been reported to enhance antitumor immune responses across various preclinical models [23].

This study has several limitations. The retrospective and single-center design, together with the relatively small sample size and a heterogeneous patient population comprising different treatment lines and immunotherapy agents, are factors limiting both the statistical power and the generalizability of the study. PD-L1 data were not available in 20.6% of patients, and the PD-L1-negative subgroup was limited to only 38 patients; therefore, this finding needs to be validated prospectively in larger cohorts. The 25% cut-off value used was determined by cohort-specific Cox regression analysis and requires external validation, as a standardized threshold for EZH2 has not yet been defined. Independent pre-consensus scores from the three pathologists were not retained, so formal interobserver agreement could not be quantified. Sparse histological categories also yielded somewhat imprecise Cox estimates. Finally, as with most real-world cohorts, the study lacked an untreated or non-ICI comparator group, which limits our ability to fully distinguish prognostic from treatment-predictive effects.

## 5. Conclusions

EZH2 is an important transcriptional regulator and an epigenetic and immunological modulator that plays a broad role in carcinogenesis. Although EZH2 expression was not found to be an independent prognostic factor in this study, the numerically favorable trends observed in EZH2-low patients across several endpoints, together with preclinical evidence linking EZH2 to immune evasion, provide a rationale for further investigation rather than a basis for clinical application at this stage. These exploratory findings should not be interpreted as evidence of predictive utility. Prospective, multicenter studies with standardized EZH2 scoring and prespecified thresholds are needed to clarify whether EZH2 provides information complementary to PD-L1 in predicting response to immunotherapy.

## Figures and Tables

**Table 1:** Patient characteristics (*n* = 102).

Variable	*n* (%)	Variable	*n* (%)
Age, median (range)	63 (41–82)	ECOG Performance Status	
		ECOG 0	59 (57.8%)
		ECOG 1	43 (42.2%)
Sex		Histology	
Male	88 (86.3%)	Adenocarcinoma	54 (52.9%)
Female	14 (13.7%)	Squamous cell carcinoma	40 (39.2%)
		Others	8 (7.9%)
Stage at Diagnosis		Smoking Status	
Metastatic	67 (65.7%)	Never smoker	7 (6.9%)
Non-metastatic	35 (34.3%)	Former smoker	50 (49.0%)
		Current smoker	43 (42.2%)
PD-L1 Expression (TPS)		Unknown/Missing	2 (1.9%)
<1%	38 (37.3%)	EZH2 Expression	
1–49%	27 (26.5%)	<25%	56 (54.9%)
≥50%	16 (15.7%)	≥25%	46 (45.1%)
Unknown/Missing	21 (20.6%)		

Abbreviations: ECOG, Eastern Cooperative Oncology Group; PD-L1, programmed death-ligand 1; TPS, tumor proportion score; EZH2, enhancer of zeste homolog 2.

**Table 2:** Treatment characteristics and immunotherapy exposure in patients with non-small cell lung cancer (*n* = 102).

Variable	*n* (%)	Variable	*n* (%)
Line of Immunotherapy		Treatment Regimen	
First-line	18 (17.6%)	Immunotherapy alone	91 (89.2%)
Second-line	74 (72.5%)	Chemoimmunotherapy	11 (10.8%)
Third-line	10 (9.8%)		
Immunotherapy Agents		Thoracic Radiotherapy	
Nivolumab (monotherapy)	88 (86.3%)	Yes	32 (31.4%)
Nivolumab + Ipilimumab	5 (4.9%)	No	70 (68.6%)
Pembrolizumab	6 (5.9%)		
Atezolizumab	3 (2.9%)		

**Table 3:** Objective response and disease control according to EZH2 expression (25% threshold).

Outcome	EZH2-High ≥ 25% (*n* = 46)	EZH2-Low < 25% (*n* = 56)	Fisher’s Exact *p*
Objective response	21 (45.7%)	30 (53.6%)	0.551
No objective response	25 (54.3%)	26 (46.4%)	
Disease control	28 (60.9%)	36 (64.3%)	0.837
No disease control	18 (39.1%)	20 (35.7%)	

Abbreviations: EZH2, enhancer of zeste homolog 2. Percentages use the total number of patients within each EZH2 group as the denominator.

**Table 4:** Exploratory early progression according to EZH2 expression in PD-L1-negative patients (*n* = 38).

	EZH2-High ≥ 25% (*n* = 13)	EZH2-Low < 25% (*n* = 25)	OR (95% CI)	Unadjusted *p*	Bonferroni-Adjusted *p*
Early progression (≤3 months)	1 (7.7%)	3 (12.0%)	0.611 (0.057–6.537)	1.000	1.000
No early progression	12 (92.3%)	22 (88.0%)			

Abbreviations: OR, odds ratio; CI, confidence interval; EZH2, enhancer of zeste homolog 2; PD-L1, programmed death-ligand 1. Percentages use each EZH2 subgroup as the denominator. Adjusted *p*-values use a Bonferroni correction across four exploratory early-progression comparisons.

**Table 5:** Multivariable Cox regression analyses (25% EZH2 threshold).

Variable	Comparison	OS HR (95% CI)	OS *p*	PFS HR (95% CI)	PFS *p*
EZH2 expression	High vs. low	0.953 (0.533–1.704)	0.870	0.996 (0.573–1.733)	0.989
ECOG	1 vs. 0	1.334 (0.746–2.386)	0.331	1.278 (0.713–2.289)	0.410
Squamous carcinoma	vs. adenocarcinoma	2.580 (1.370–4.857)	0.003	1.888 (1.066–3.346)	0.029
NOS	vs adenocarcinoma	6.886 (1.401–33.857)	0.018	3.927 (0.852–18.099)	0.079
Carcinosarcoma	vs adenocarcinoma	6.044 (0.727–50.230)	0.096	13.565 (1.563–117.707)	0.018
PD-L1	Negative vs. positive	1.258 (0.659–2.400)	0.487	1.237 (0.684–2.238)	0.482
Treatment line	Second vs. first	0.927 (0.376–2.286)	0.869	1.170 (0.520–2.629)	0.704
Treatment line	Third vs. first	0.309 (0.087–1.092)	0.068	0.715 (0.244–2.094)	0.541

Abbreviations: HR, hazard ratio; CI, confidence interval; ECOG, Eastern Cooperative Oncology Group; EZH2, enhancer of zeste homolog 2; PD-L1, programmed death-ligand 1; NOS, not otherwise specified. Reference categories were EZH2-low, ECOG 0, adenocarcinoma, PD-L1-positive, and first-line treatment. One rare histological category with no OS events was non-estimable and is not displayed. Models used 81 complete cases because PD-L1 was unavailable for 21 patients.

**Table 6:** Sensitivity analyses using 25% and 50% EZH2 thresholds.

Outcome	25% Threshold: Low vs. High	*p*	50% Threshold: Low vs. High	*p*
ORR	53.6% vs. 45.7%	0.551	53.8% vs. 37.5%	0.243
DCR	64.3% vs. 60.9%	0.837	65.4% vs. 54.2%	0.343
Early progression	7.1% vs. 17.4%	0.132	9.0% vs. 20.8%	0.147
Median OS	37 vs. 27 months	0.323	33 vs. 26 months	0.228
Median PFS	15 vs. 12 months	0.387	15 vs. 9 months	0.295
Adjusted OS HR, high vs. low	0.953 (0.533–1.704)	0.870	0.897 (0.441–1.825)	0.765
Adjusted PFS HR, high vs. low	0.996 (0.573–1.733)	0.989	1.039 (0.524–2.060)	0.912

Abbreviations: ORR, objective response rate; DCR, disease control rate; OS, overall survival; PFS, progression-free survival; HR, hazard ratio. ORR, DCR, and early-progression *p*-values are from two-sided Fisher’s exact tests; survival *p*-values are from log-rank tests. Adjusted HRs are from treatment-line-adjusted multivariable Cox models.

## Data Availability

The datasets generated and/or analysed during the current study are not publicly available due to patient privacy considerations but are available from the corresponding author on reasonable request.
